# Localization of general and regulatory proteolysis in *Bacillus subtilis* cells

**DOI:** 10.1111/j.1365-2958.2008.06438.x

**Published:** 2008-09-29

**Authors:** Janine Kirstein, Henrik Strahl, Noël Molière, Leendert W Hamoen, Kürşad Turgay

**Affiliations:** 1 Institut für Biologie – MikrobiologieFU Berlin, Königin-Luise-Str. 12-16, 14195 Berlin, Germany; 2 Institute for Cell and Molecular Biosciences, Newcastle UniversityFramlington Place, Newcastle NE2 4HH, UK

## Abstract

Protein degradation mediated by ATP-dependent proteases, such as Hsp100/Clp and related AAA+ proteins, plays an important role in cellular protein homeostasis, protein quality control and the regulation of, e.g. heat shock adaptation and other cellular differentiation processes. ClpCP with its adaptor proteins and other related proteases, such as ClpXP or ClpEP of *Bacillus subtilis*, are involved in general and regulatory proteolysis. To determine if proteolysis occurs at specific locations in *B. subtilis* cells, we analysed the subcellular distribution of the Clp system together with adaptor and general and regulatory substrate proteins, under different environmental conditions. We can demonstrate that the ATPase and the proteolytic subunit of the Clp proteases, as well as the adaptor or substrate proteins, form visible foci, representing active protease clusters localized to the polar and to the mid-cell region. These clusters could represent a compartmentalized place for protein degradation positioned at the pole close to where most of the cellular protein biosynthesis and also protein quality control are taking place, thereby spatially separating protein synthesis and degradation.

## Introduction

Protein degradation plays an important role in protein homeostasis and in protein quality control. Together with molecular chaperone systems, proteases ensure the proper function of proteins in their cellular environment (Hartl and Hayer-Hartl, 2002; Bukau *et al*., 2006). Proteolysis is also used for regulatory purposes, such as the regulated degradation of key transcription factors that control cell cycle, developmental or adaptation processes (Ciechanover, 1998; Gottesman, 2003; Jenal and Hengge-Aronis, 2003; Pickart and Cohen, 2004).

Both general and regulatory proteolysis are mediated by dedicated molecular machines, which are ATP-dependent proteases consisting of ring-forming hexameric Hsp100/Clp proteins of the AAA+ family, associated on both sides of the barrel-forming peptidases complex. The AAA+ proteins of the protease can recognize, unfold and translocate proteins into the proteolytic chamber of the interacting peptidase compartment (Lupas *et al*., 1997; Wickner *et al*., 1999; Pickart and Cohen, 2004; Sauer *et al*., 2004). An example of such proteases in eukaryotes is the proteasome (Ciechanover, 1998; Pickart and Cohen, 2004). In prokaryotic cells, homologous protease complexes are formed by the AAA+ proteins ClpA, ClpX or ClpC, which assemble with the peptidase ClpP (Wickner *et al*., 1999; Sauer *et al*., 2004). Relatively little is known concerning the subcellular localization of these protease systems, their substrates and other components of the protein quality control system (Wojcik and DeMartino, 2003; Lindner *et al*., 2008), even though this information is important for a full comprehension of the diverse and important intracellular processes involving proteolysis and protein quality control (Balch *et al*., 2008).

We use the model organism *Bacillus subtilis* to investigate the dynamic subcellular localization of the Hsp100/Clp protease systems. Cells of a *B. subtilis* population can adapt and react to a wide array of environmental changes by, e.g. heat shock and general stress adaptation (Hecker *et al*., 2007), and developmental processes, such as sporulation (Rudner and Losick, 2001) or competence development (Chen *et al*., 2005).

The *B. subtilis* Hsp100/Clp proteins ClpC, ClpE and ClpX associate with ClpP to form protease complexes. Of these three Hsp100/Clp proteins, ClpX is the most abundant Clp ATPase under normal growth conditions (Gerth *et al*., 2004). ClpC and ClpX are intricately involved in both general protein quality control (Krüger *et al*., 1994; 2000; Wiegert and Schumann, 2001; Schlothauer *et al*., 2003; Kock *et al*., 2004; Zellmeier *et al*., 2006; Turgay, 2007) and various processes, such as the regulation of developmental and adaptation processes (Liu *et al*., 1999; Liu and Zuber, 2000; Pummi *et al*., 2002; Zuber, 2004; Mascarenhas *et al*., 2005; Ruvolo *et al*., 2006).

There are specific adaptor proteins that enable or enhance the targeting of specific and general substrates to the protease systems (Dougan *et al*., 2002). For example, the tyrosine kinase McsB is an adaptor protein that binds specifically the heat shock regulator CtsR and targets it for degradation by the ClpCP protease. As such, McsB is responsible for the heat-induced inactivation and subsequent degradation of the repressor protein CtsR, thereby controlling the induction of the class III heat shock regulon that also encompasses the *clpC* operon, *clpP* and *clpE*, in *B. subtilis* (Krüger *et al*., 2001; Kirstein *et al*., 2005; 2007). For ClpC, several adaptor proteins have been identified (Turgay *et al*., 1998; Persuh *et al*., 2002; Schlothauer *et al*., 2003; Kirstein *et al*., 2006; 2007). As a further regulatory element, the activity of adaptor proteins, such as MecA and McsB, can itself be subject to regulation. For example, only the phosphorylated form of McsB binds CtsR, and the autophosphorylation activity of McsB is regulated by the protein McsA (Kirstein *et al*., 2007; Prepiak and Dubnau, 2007).

Biochemical experiments have shown that the adaptor proteins are assisting the oligomerization of the active ClpC hexamer, which is a precondition for the assembly of the ClpP oligomer and the formation of the whole ClpCP protease complex (Kirstein *et al*., 2006). Different adaptor proteins, with their substrates, can compete for ClpC (Kirstein *et al*., 2007).

It is well possible that the different Clp-dependent processes occur while the different components diffuse freely through the cytoplasmic space. However, localization and activation of the specific protease complexes within the cell could contribute to the control and separation of the different processes involving proteolysis (Kirstein *et al*., 2006; 2007). There are now many examples of proteins that localize at specific subcellular positions in the bacterial cell (Ebersbach and Jacobs-Wagner, 2007; Morris and Jensen, 2008), including ClpXP from *Caulobacter crescentus* (McGrath *et al*., 2006). We were interested to know whether this could also be the case for the Hsp100/Clp proteases of *B. subtilis*. Therefore, we analysed the cellular localization of the Clp system, the adaptor protein McsB, its substrate CtsR (Kirstein *et al*., 2007) and aggregated heterologous proteins forming inclusion bodies representing general substrates (Nurminen *et al*., 1992; Jürgen *et al*., 2001).

The results of these experiments demonstrated that the ATPases and the proteolytic subunit of the Clp proteases are forming dynamic clusters at the polar regions of the cell. The localization of ClpP appeared to depend on the presence of the ATPase. Moreover, the adaptor protein McsB and its substrate CtsR, as well as aggregated proteins forming inclusion bodies, displayed similar localization patterns as shown for the proteases. Our experiments suggest that the polar region is an important subcellular site for compartmentalized protein degradation in *B. subtilis*.

## Results

### Localization of ClpP, ClpX, ClpC and ClpE

To examine the distribution of Clp proteins *in vivo*, we constructed C-terminal fusions of ClpP, ClpC, ClpE and ClpX to GFP or its fluorescent variants (Table 1). Fusion of GFP at the C-terminus allows normal transcriptional and translational regulation. As the fusions were introduced by Campbell integration, the original gene has been replaced by the *gfp* fusion and thus represents the only active copy of the respective gene. Deletions of *clpP*, *clpX* and *clpC* have very pleiotropic effects, and especially in the case of *clpP* and *clpX*, clearly detectable phenotypes in growth and appearance (Gerth *et al*., 1998; Msadek *et al*., 1998; Pan *et al*., 2001). All these constructed fusion strains grew normally, indicating that the fusion to GFP did not impair their functionality. Western blot experiments confirmed the presence of only single intact GFP-tagged proteins in these strains. In addition, the heat shock induction of McsB and CtsR was comparable with that of a wild-type strain (data not shown and Fig. S4).

**Table 1:** List of strains used in this study.

Strains	Relevant genotype/properties	Source/construction
*B. subtilis* 168	*trpC*2	Anagnostopoulos and Spizizen (1961)
QBP418	Δ*clpC*::*tet*	Pan *et al*. (2001)
BEK90	Δ*clpX*::*kan*	Gerth *et al*. (2004)
BUG1	Δ*clpP*::*spc*	Gerth *et al*. (2004)
BMM01	Δ*ywlE*::*spc*	Gift from Ulf Gerth (Uni Greifswald)
ClpC-DWB	*clpC-DWB* (ClpCE280A/E618A)	Kirstein *et al*. (2006)
JK01	*cat clpC–gfp*	This work (see M and M)
JK02	*cat clpC-cfp*	This work (see M and M)
JK03	*cat clpP–gfp*	This work (see M and M)
JK04	*cat clpP–yfp*	This work (see M and M)
JK05	*cat clpC–yfp*	This work (see M and M)
JK06	*cat clpX–yfp*	This work (see M and M)
JK07	*spc clpC–cfp*	This work (see M and M)
JK08	*cat mcsB–yfp*	This work (see M and M)
JK09	*cat ctsR–gfp*	This work (see M and M)
JK10	*cat spc clpP–yfp clpC–cfp*	This work (see M and M)
JK11	*cat spc clpP–yfp clpX–cfp*Δ*clpX*::*kan*	This work (see M and M)
JK12	*cat clpP–gfp*Δ*clpX*::*kan*	This work [chr. DNA (BEK90) moved into JK03]
JK13	*cat clpX–yfp*Δ*clpP*::*spc*	This work [chr. DNA (BUG1) moved into JK06]
JK14	*cat clpP–gfp*Δ*clpC*::*tet*	This work [chr. DNA (QBP418) moved into JK03]
JK15	*cat clpC–gfp*Δ*clpP*::*spc*	This work [chr. DNA (BUG1) moved into JK02]
JK16	*cat mcsB–yfp*Δ*clpC*::*tet*	This work [chr. DNA (QBP418) moved into JK08]
JK17	*cat mcsB–yfp*Δ*ywlE*::*spc*	This work [chr. DNA (BMM01) moved into JK08]
JK18	*cat ctsR–gfp clpC-DWB*	This work [chr. DNA (ClpC-DWB) moved into JK09]
JK19	*cat clpE–yfp*	This work
IH7282	IH6140 (pKTH1784)	Gift from V. Kontinen (National Public Health Institute, Helsinki, Finland) Jürgen *et al*. (2001)
IH6627	IH6140 (pKTH290)	Gift from V. Kontinen (National Public Health Institute, Helsinki, Finland) Nurminen *et al*. (1992)
IH6140	Low exoprotease activity strain derived from *B. subtilis* Marburg 6064	Palva *et al*. (1983)
JK20	IH6627 *cat clpP–gfp*	This work (see M and M)
JK21	IH7282 *cat clpP–gfp*	This work (see M and M)
JK22	IH6627 *cat clpC–gfp*	This work (see M and M)
JK23	IH7282 *cat clpC–gfp*	This work (see M and M)
JK24	IH6627 *cat mcsB–yfp*	This work (see M and M)
JK25	IH7282 *cat mcsB–yfp*	This work (see M and M)
JK26	IH6627 *cat clpE–yfp*	This work (see M and M)
JK27	IH7282 *cat clpE–yfp*	This work (see M and M)
1801	*trpC2 chr::pJSIZDpble (Pspac–ftsZ ble)*	*B. subtilis* 186 transformed with pJSIZDpble Beall and Lutkenhaus (1991)
PL20	*erm chr*::*pJS1* (*Pspac–dnaA*)	Prolysis, Yarnton, Oxford, UK
HS10	*ble cat Pspac-ftsZ clpP–gfp*	This work (see M and M)
HS11	*erm cat Pspac–dnaA clpP–gfp*	This work (see M and M)
HS12	*spc amyE*::*clpX–cfp*Δ*clpX*::*kan*	This work (see M and M)

#### ClpP

**Fig. 1:**
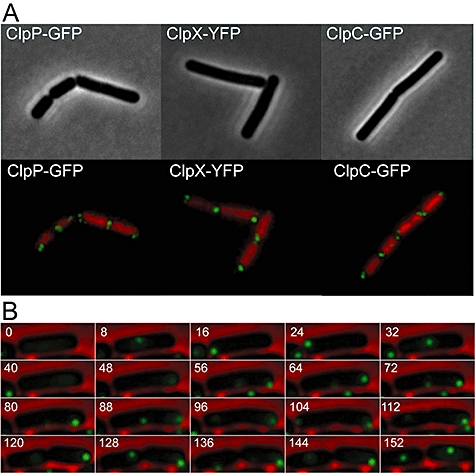
Subcellular localization of the Clp proteins. A. Upper panel: phase contrast images of *B. subtilis* strains encoding ClpP–GFP, ClpX–YFP and ClpC–GFP cultivated in LB medium at 30°C. Lower panel: overlay of the GFP signal of the fusion proteins (pseudocoloured in green) and the nucleoid stained with DAPI (pseudocoloured in red). B. Dynamic localization of ClpP–GFP. A selected field of the time-lapse movie (see Movie S2) with pictures taken every 8 min as indicated in the top left corner of the respective picture (See *Experimental procedures* for details).

We first examined the localization of ClpP–GFP in *B. subtilis* cells. The *clpP–gfp* fusion strain revealed clear fluorescent foci (Fig. 1). We analysed the localization patterns of the Clp–GFP proteins in more than 200 cells. The results are summarized in Table 2 and indicate that the detected fluorescent foci were present in almost all cells and mostly localized in vicinity to the cell poles or the mid-cell region.

**Table 2:** Analysis of the number and localization of foci of the GFP fusion proteins at 30°C and after a 10 min heat shock at 50°C.

	*clpP–gfp*		*clpC–gfp*		*clpX–gfp*		*clpE–gfp*	*mcsB–gfp*	*ctsR–gfp*
						
	30°C	50°C	30°C	50°C	30°C	50°C	50°C	50°C	50°C
Average number of foci per cell	1.4	4.5	0.59	3.6	0.77	3.2	3.6	3.0	3.1
Average number of foci located close to the pole	1.15	2.6	0.54	2.6	0.72	2.1	2.1	2.2	2.0
Average number of foci located close to mid-cell	0.17	1.4	0.04	1.4	0.05	1.2	1.2	1.1	1.2
% of cells that display also mid-cell located foci	17	79	5	73	5	70	62	55	71
% of cells that display also quarter-cell located foci	7	56	0	32	0.5	21	25	19	19

#### ClpX, ClpC and ClpE

As the Clp ATPases form functional proteolytic complexes with ClpP, the question arises where these proteins are located in the cell. As shown in Fig. 1A and analysed in Table 2, the foci detected in a strain containing a *clpC–gfp* fusion were less abundant (0.59 foci/cell) than in the *clpP–gfp* strain (1.4 foci/cell). Nevertheless, the relative distribution of fluorescent foci appeared quite similar to the distribution found for ClpP–GFP. Examining a ClpX–YFP fusion, we observed more abundant discrete foci (0.77 foci/cell) compared with ClpC, but less compared with ClpP, in patterns similar to those found for ClpP and ClpC (Fig. 1A and Table 2). These results reflects the previously determined relative number of ClpP, ClpX and ClpC in *B. subtilis* cells (Gerth *et al*., 2004). ClpE, which is the most strictly controlled *clp* gene, harbouring five CtsR binding sites within its promoter region, could not be detected under these growth conditions (Gerth *et al*., 2004).

In conclusion, the peptidase moiety of the Clp protease complexes, ClpP, and its associated ATPases ClpX and ClpC are not equally distributed over the cell under normal growth conditions, but assemble into clusters in proximity to the pole or close to mid-cell where, upon cell division, the new poles of the daughter cells are formed.

### Dynamic localization of ClpP

To follow the dynamic subcellular localization of the ClpP–GFP fusion proteins, we observed the outgrowth of a single *B. subtilis clpP–gfp* cell into a microcolony in chemically defined medium using time-lapse fluorescence microscopy (Veening *et al*., 2008). Pictures were taken every 8 min for more than 12 h. The result of this experiment is presented as a movie of a whole growing colony and an additional movie detailing single cells of that growing colony (Movies S1 and S2). The result of the time-lapse experiment is also depicted as frames of a representative detail in Fig. 1B.

The localization of ClpP was analysed in cells of three independent time-lapse experiments at two different time points (a total of about 1300 cells). In addition, 280 individual cell divisions from three independent time-lapse experiments were analysed for presence and localization of ClpP foci. A ClpP focus is visible in 70–80% of all cells at any given time point. However, 96% of all cells going through cell division appear to have a ClpP focus. About 89% of the cells feature a ClpP focus close to the cell division site, and 21% have an additional focus localized elsewhere in the cell mostly at the old cell pole. These observations suggest that under the tested growth conditions, polar Clp–GFP foci often originate at mid-cell and subsequently end up at the cell poles.

The time-lapse movies suggest that fluorescent foci disappear and appear at different positions, suggesting that the foci formation is reversible and not fixed. It does not appear as if the foci themselves move or are mobile. To observe the ClpP foci dynamics at a higher time resolution, we repeated the time-lapse with shorter time intervals and took images every minute over a 90 min time period (Movie S3). In this experiment, the cells were cultured in LB medium instead of chemically defined medium in order to achieve a faster growth rate. The observed localization and the ability of the ClpP cluster to form and dissolve in the polar and mid-cell region of the *B. subtilis* cells (Fig. 1B and Movies S1 and S2) were confirmed.

### Localization upon heat shock

**Fig. 2:**
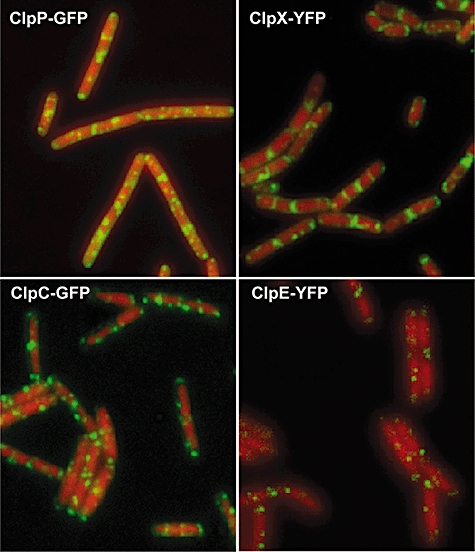
Localization of the Clp proteins upon heat schock. Cellular localization of ClpP–GFP, ClpC–GFP, ClpX–YFP and ClpE–YFP after heat shock at 50°C. The images show an overlay of the GFP/YFP signal of the fusion proteins (pseudocoloured in green) and the nucleoid stain DAPI (pseudocoloured in red).

As the synthesis of Clp proteins is induced upon stress, such as heat shock or oxidative stress (Gerth *et al*., 2004), we studied the localization of Clp proteins after a 10 min incubation at 50°C. The localization patterns of ClpP and the Clp ATPases ClpC and ClpX in these heat-shocked cells did not change substantially, except that the fluorescent signals became much stronger and additional foci appeared (Fig. 2). In addition, the localization pattern of ClpP was confirmed by immuno-fluorescence microscopy (data not shown). Under heat shock conditions, ClpE–GFP foci became visible as well, and showed a localization pattern reminiscent of that of the other ATPases (Fig. 1).

The localization patterns of the Clp proteins after heat shock were quantitatively analysed and the results are summarized in Table 2. All cells contained at least one Clp protein cluster, and in cells where only a single cytoplasmatic focus was detected, it was always localized at the pole. The average number of foci per cell ranged from 3.2 (ClpX) to 4.5 (ClpP). The number as well as the subcellular distribution of foci was very similar for ClpP and the ATPases, ClpC, ClpE and ClpX. On average, 2.1 foci for ClpE and ClpX, and 2.6 foci for ClpC and ClpP were localized at the poles. Cells containing two foci positioned these close to the poles and at mid-cell.

The distribution and number of foci in *clpP–gfp* cells, which were immediately returned to growth at 30°C after heat shock, appeared, 1 or 2 h after the heat shock, similar to non-heat-shocked cells. We could detect no significant difference between the localization pattern of heat-shocked *clpP–gfp* cells with or without a pre-adaptive 42°C heat shock (data not shown). When we inhibited new protein biosynthesis prior to the heat shock (using high concentrations of the translation inhibitor chloramphenicol), ClpP foci formation was not prevented (Fig. S1), suggesting that the foci formation does not depend on new protein biosynthesis.

We also used diamide treatment to study the effect of oxidative stress (Leichert *et al*., 2003). This treatment resulted in similar localization patterns compared with heat-shocked cells (data not shown).

### Colocalization of ClpC, ClpX, ClpE and ClpP

**Fig. 3:**
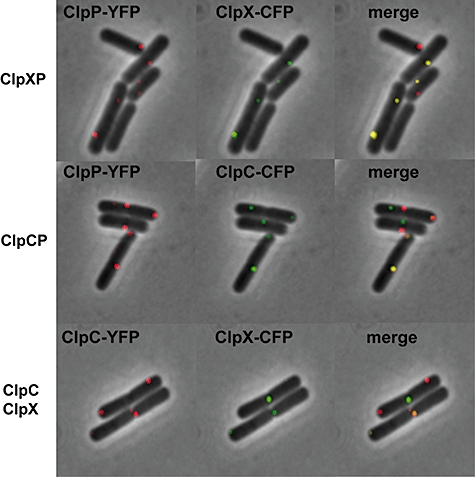
Colocalization of Clp proteases. Dual labelling of ClpX–ClpP (upper panel), ClpC–ClpP (middle panel) and ClpC–ClpX (lower panel) at 30°C. The images were obtained using YFP filters (left column) and CFP filters (middle column). An overlay of the YFP and CFP channels is shown on the right. YFP fluorescence has been pseudocoloured in red and CFP in green.

The similar localization pattern of the Clp peptidase and Clp ATPases, together with the biochemically well-established and -observed complex formation of ClpCP and ClpXP strongly suggests that the observed foci represent functional Clp protease complexes (Gerth *et al*., 2004; Kirstein *et al*., 2006). To analyse the localization of these complexes, we took a dual labelling approach and constructed strains expressing a ClpP–YFP fusion as well as a ClpX–CFP or a ClpC–CFP fusion. At 30°C we could detect overlapping foci for ClpX and ClpP or ClpC and ClpP in the respective strains (Fig. 3). In the *clpC–CFP clpP–YFP* strain we analysed 243 cells that displayed foci. These cells showed 320 ClpP foci (1.3/cell) and 183 ClpC foci (0.75/cell). Furthermore 146 (0.6/cell) of these foci were overlapping, suggesting a colocalization of ClpC and ClpP in these foci. In the *clpX–CFP* and *clpP–YFP* strain we analysed 238 cells with visible foci. These cells showed 309 ClpP foci (1.3/cell) and 169 ClpX foci (0.71/cell) and 129 overlapping ClpX and ClpP foci (0.54/cell). These results indicate a reasonable overlap of localization for ClpC, ClpX and ClpP to form functional ClpCP or ClpXP proteases localized to these foci under normal growth conditions.

We also constructed a strain carrying *clpC–YFP* and a *clpX–CFP* fusion and analysed 374 cells with visible foci (Fig. 3). In these cells we counted 289 ClpC foci (0.77/cell), 268 ClpX foci (0.77/cell) and 107 (0.29/cell) of these foci were overlapping in localization. This observed colocalization of about a third of the foci suggests that both ClpCP and ClpXP proteases can be present in the same clusters.

#### Heat shock

We observed a strong colocalization of ClpX and ClpP or ClpC and ClpP under heat shock conditions. A hundred cells with foci were counted. In the *clpX–CFP clpP–YFP* strain 298 ClpX foci (3.9/cell) and 441 ClpP foci (4.4/cell) with 241 (2.4/cell) overlapping foci were detected. For the *clpC–CFP clpP–YFP* strain 342 (3.4/cell) ClpC and 462 ClpP (4.6/cell) foci with 278 colocalizing foci (2.8/cell) were counted. ClpE, for which foci are only detectable under heat shock conditions colocalized strongly (about 70%) with ClpC or ClpX (Fig. S2).

These data suggest that the Clp proteases localize to specific confined spaces within the bacterial cells, primarily in the area close to the cell poles. Under normal growth conditions both ClpCP and/or ClpXP protease species can constitute these clusters. When heat shock was applied more protease clusters or higher order structure of assembled proteases formed. ClpE, most probably also assembling to ClpEP protease complexes (Gerth *et al*., 2004; Miethke *et al*., 2006), appeared to become part of these protease clusters, too.

### ATPase controlled localization of ClpP

For the ClpCP protease, we have previously demonstrated that the activation of ClpP requires the association with the hexameric ClpC ATPase complex. The interaction with the oligomeric ATPase subsequently facilitates the assembly of the whole proteolytic complex (Kirstein *et al*., 2006). We wanted to investigate whether this ATPase-dependent activation of ClpP is required for the subcellular localization and clustering of ClpP. To test this, we examined the ClpP–GFP distribution in a *clpX* and *clpC* background.

**Fig. 4:**
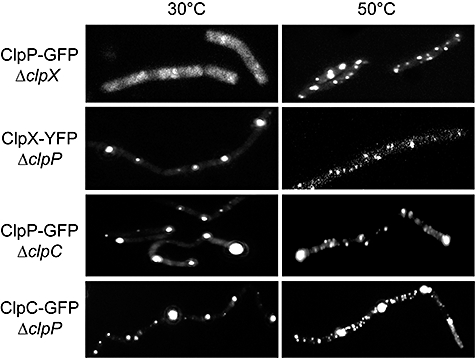
Analysis of Clp proteins in different mutant strains. Cellular localization of ClpP–GFP in a Δ*clpX* background, ClpX–YFP in a Δ*clpP* background, ClpP–GFP in a Δ*clpC* background and ClpC–GFP in a Δ*clpP* background, before (30°C) and after heat shock (50°C).

The images depicted in Fig. 4 demonstrate that the localization of ClpP–GFP becomes diffuse when ClpX is absent. After heat shock the *clpX* mutant showed normal ClpP–GFP foci again (Fig. 4). Upon heat shock the two stress-inducible ATPases, ClpC and ClpE, reach considerably higher concentrations in the cell (Gerth *et al*., 2004). This suggests that the delocalization of ClpP in a *clpX* mutant can be compensated by the raised amounts of ClpC and ClpE proteins under heat shock conditions. As shown in Fig. 4, the absence of ClpC did not result in a delocalization of ClpP, neither at 30°C nor at 50°C. Even a double knockout of *clpC* and *clpE* did not result in a delocalization of ClpP (data not shown). Although ClpX is not stress-inducible, its high basal protein level seems to be sufficient to promote the localization of ClpP after heat shock in the absence of the other ATPases. On the other hand, the localization of ClpX and the two other Clp ATPases did not depend on the presence of ClpP (Fig. 4 and data not shown), which is in agreement with our *in vitro* model of a hierarchical assembly of the Clp proteases (Kirstein *et al*., 2006). We conclude that the localization of ClpP to the active protease complex is determined by the ATPase component.

### Localization of adaptor and substrate proteins

If the large ClpCP protease clusters that assemble at the cell poles are active, then the localization of adaptor proteins and substrates should show a similar localization pattern under conditions when proteolysis is taking place. We chose the well-characterized adaptor McsB and its substrate CtsR to study the localization of an adaptor protein and its substrate. In a previous study, we have shown that the tyrosine kinase McsB acts only in its active phosphorylated state as an adaptor protein that targets CtsR for degradation by ClpCP. The McsB kinase activity and phosphorylation state is positively regulated by its activator protein McsA, and can be inhibited by ClpC and the tyrosine phosphatase YwlE. This regulatory circuitry enables *B. subtilis* cells to both sense and respond to heat shock by controlling the activity and stability of the repressor CtsR (Kirstein *et al*., 2005; 2007).

**Fig. 5:**
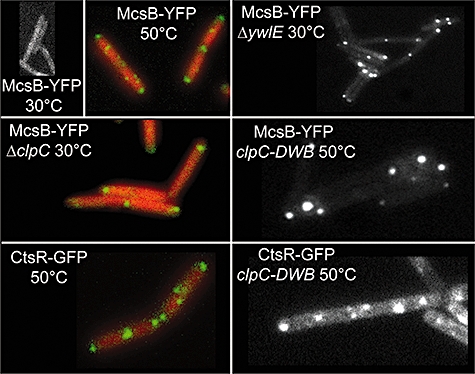
Analysis of Clp protease adaptor and substrate localization in different mutant strains. Cellular localization of adaptor McsB (McsB–YFP) in *wild type*, Δ*clpC*, Δ*ywlE* or *clpC-DWB* background and substrate CtsR (CtsR–GFP) in wild type or a *clpC*-*DWB* background, before (30°C) or after heat shock (50°C).

We constructed a McsB–YFP fusion strain as described before. Western blot experiments with anti-McsB and anti-GFP antibodies demonstrated that only a single YFP-tagged McsB could be detected in this strain, and the heat shock induction of McsB and CtsR in the *mcsB–yfp* strain was comparable with wild type (data not shown and Fig. S4). Under normal growth conditions only a weak and diffuse fluorescence signal was observed (Fig. 5). The translation of *mcsB* and *clpC* is coupled (Krüger *et al*., 2001), and upon heat shock, when McsB fulfils its adaptor function, polar foci of McsB were clearly detectable (Fig. 5 and Table 1).

As mentioned before, the kinase activity of McsB is kept inactive by ClpC and counteracted by the specific phosphatase YwlE (Kirstein *et al*., 2005). When a *clpC* or an *ywlE* deletion was introduced into the *mcsB–yfp* fusion strain, McsB displayed a heat shock-like localization pattern already at 30°C (Fig. 5).

We conclude from these data that the McsB clusters, which assemble and localize after heat shock, represent the phosphorylated, adaptor-active form of McsB.

The question that remains is whether the substrate CtsR is also localized to the protease clusters. We constructed a strain where CtsR was replaced by CtsR–GFP. Western blot experiments with anti-CtsR and anti-GFP antibodies demonstrated that only a single GFP-tagged CtsR could be detected in this strain and the heat shock induction of McsB and CtsR in the *ctsR–gfp* strain was comparable with the wild-type strain (data not shown).

At 30°C no clear localization pattern of CtsR–GFP could be detected but upon heat shock, when CtsR is targeted for degradation, CtsR localized preferentially to the cell pole and mid-cell, thus resembling the localization pattern of McsB, ClpC and ClpP (Fig. 5, see also Table 1). Previously, we have demonstrated that mutations in the Walker-B motives of ClpC result in a protein (ClpC-DWB) that forms a stable substrate interaction complex with its adaptor and substrate proteins (Kirstein *et al*., 2006). Western blot experiments confirmed that both McsB–YFP and CtsR–GFP were stabilized in the *clpC-DWB* mutant background (data not shown and Fig. S4). As depicted in Fig. 5, the localization pattern of McsB and CtsR did not appear to change in the presence of the ClpC-DWB variant, suggesting that the stabilized ‘trapped’ substrate-delivery-ClpCP-complex localizes in a similar way compared with the wild-type situation. These data support the assumption that ClpCP-mediated degradation is confined to a specific space in the cytoplasm of *B. subtilis*.

### Effect of inclusion bodies on protease localization

As a second system to examine the *in vivo* localization of substrate proteins and the protease system, we took advantage of a well-characterized *B. subtilis* strain that expresses the outer membrane protein PorA from *Neisseria meningitides* yielding intracellular aggregates (Nurminen *et al*., 1992). Using electron microscopy and immuno-gold labelling, it was shown that ClpC, ClpP and ClpX associate with these PorA inclusion bodies (Nurminen *et al*., 1992; Jürgen *et al*., 2001). Association of ClpCP and ClpXP with protein aggregates has also been observed in heat-shocked *B. subtilis* cells (Krüger *et al*., 2000). Further analysis demonstrated that the overexpression of PorA resulted in a cellular stress response and upregulation of the transcription of genes encoding chaperones and the Clp proteins, similar to a heat shock stress response in *B. subtilis* (Jürgen *et al*., 2001).

**Fig. 6:**
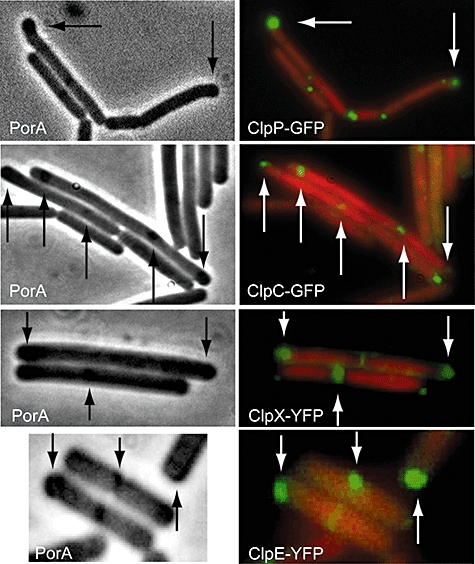
Colocalization of ClpP, ClpX, ClpC and ClpE with inclusion bodies. Inclusion bodies were visualized by phase contrast microscopy (left panel) and the different GFP/YFP fusions were visualized by fluorescence microscopy (right panel). Arrows indicate inclusion bodies visible in phase contrast images.

We transferred the different GFP or YFP fusions into a PorA-overexpressing *B. subtilis* strain. In these cells inclusion bodies were easily detected by phase contrast microscopy and almost always located in close proximity to the poles or cell division sites (Fig. 6). When the strains were analysed with fluorescence microscopy, it became apparent that the Clp proteins colocalize with the PorA protein aggregates. As overexpression of PorA induces a heat shock-like stress response in *B. subtilis*, ClpE foci became visible and localized to the inclusion bodies as well (Fig. 6).

### ClpP clustering is independent of cell division and changes when replication is stalled

**Fig. 7:**
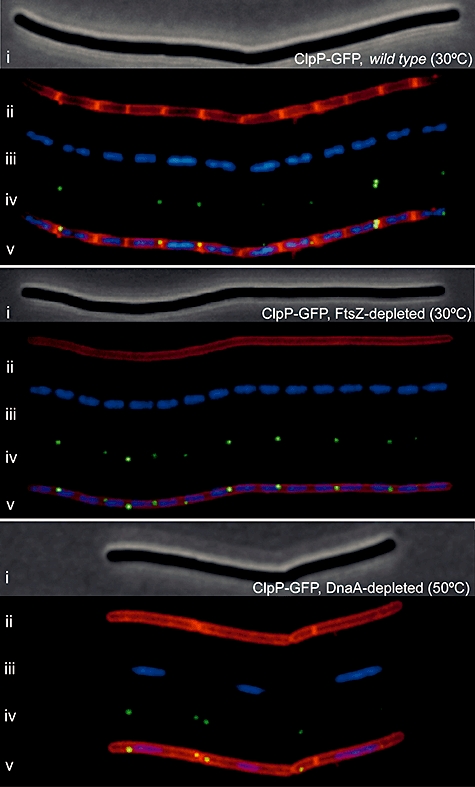
Cellular localization of ClpP in the absence of cell division and replication. The ClpP localization is visualized in wild type (upper panel), and FtsZ-depleted *B. subtilis* strains at 30°C (middle panel), and in a DnaA-depleted *B. subtilis* strain at 50°C (lower panel). Phase contrast (i), membrane stain (ii), nucleoid stain (iii), GFP fluorescence (iv) and the merged images (v) are subsequently depicted (see *Experimental procedures* for details).

Under normal growth conditions the clustering of ClpP proteases depends on the ATPase ClpX. ClpX has been implicated in the regulation of septum synthesis as this chaperone represses the polymerization of FtsZ the first step in cell division (Weart *et al*., 2005). To examine the effect on cluster formation when this essential cell division protein is absent, we introduced the ClpP–GFP fusion into a strain containing an inducible copy of *ftsZ* (*Pspac–ftsZ*). In the absence of inducer (IPTG in this case), such cells form long filaments due to the depletion of FtsZ. As shown in Fig. 7, under those conditions ClpP foci are still formed, indicating that the clustering of ClpP does not depend on cell division. Interestingly, the ClpP foci were regularly spaced over the filamentous cells. 4,6-diamino-2-phenylindole (DAPI) staining showed that the ClpP foci were located between nucleoids, suggesting that the large ClpP protease clusters are excluded from the space occupied by the nucleoids. To test this further, we examined ClpP localization after blocking of chromosome replication using a strain containing an IPTG-inducible *dnaA* gene (*Pspac–dnaA*). Depletion of DnaA, a protein essential for the initiation of chromosome replication, results in elongated cells with large cytosolic spaces devoid of chromosomal DNA (Ogura *et al*., 2001). Surprisingly, when cells were grown in the absence of IPTG, the ClpP foci disappeared over time (Fig. S3). However, a short heat shock was sufficient to produce multiple ClpP foci again (Fig. 7). This did not require *de novo* synthesis of proteins as the presence of chloramphenicol did not prevent this process (Fig. S3). We determined the position of roughly 200 ClpP foci (in about 60 cells) after DnaA depletion followed by heat shock. About 20% of the foci were located close to the cell poles, and about 10% of foci were located close to nucleoids. However, the majority (∼70%) of foci were randomly localized in the large cytosolic spaces devoid of chromosomal DNA, and only 2% of foci were found within nucleoids. Thus it seems that ClpP clusters can appear after heat shock in the wider cytosolic space of DnaA-depleted cells in a less restricted manner, but still excluded from the nucleoid in the central area of cells. Both results suggest that the localization of the ClpP foci appear excluded from the nucleoid area and depends on the available space.

## Discussion

We have visualized the proteolytic and ATPase components of the HSP100/Clp proteases in living cells of *B. subtilis*, and observed that they form protein clusters primarily at the polar regions of the cells. These clusters likely represent active protease complexes for the following reasons: (i) dual labelling showed colocalization of the ATPases ClpX, and ClpC with the ClpP peptidase; (ii) the clusters increase in size and number upon heat shock or oxidative stress; (iii) the model substrate CtsR with adaptor protein McsB showed a similar localization pattern to the ClpP components after heat shock, when degradation is taking place; and (iv) a *clpC-DWB* mutant traps the substrate CtsR to the cell pole upon heat shock.

During normal logarithmic growth there are about 17 000 ClpP, 8400 ClpX, 1500–3000 ClpC and 600 ClpE molecules per cell, suggesting that in normal growing cells, about 700–1000 active ClpXP proteases can be formed (Gerth *et al*., 2004).

Not all the proteases concurrently localize to these clusters, because in our microscopy studies there were always cells without clear GFP foci under non-stress conditions, and when protein synthesis was blocked the size and number of ClpP–GFP foci still increased upon heat shock. We also noticed in the time-lapse experiments with growing microcolonies that ClpP–GFP foci appeared and disappeared in single cells. In addition, blocking new protein synthesis by chloramphenicol did not prevent an increase of size and numbers of ClpP–GFP foci upon heat shock. All these results suggest that a dynamic equilibrium exists, between the clustered proteases in foci and proteases or protease components we could not detect.

Interestingly, we also observed foci that were composed of ClpCP and ClpXP proteases, but also foci, which contained only one species. When heat shock was applied ClpE strongly colocalized with both protease species, supporting the suggested role of ClpEP as a back-up protease supporting the other proteases under stress (Gerth *et al*., 2004).

A clustering of proteins that function in dedicated higher ordered multi-protein complexes detected as visible foci of fluorescent fusion proteins were described in bacteria but have also been observed in eukaryotic cells (An *et al*., 2008). In *B. subtilis* such higher order structures have been demonstrated for, e.g. replication factories (Lemon and Grossman, 1998), DNA-translocases (Burton *et al*., 2007) and non-ribosomal peptide-/polyketide-hybrid synthetases (Straight *et al*., 2007). What could be the function of clustering into large protease clusters? The spatial constraint and clustering of regulatory and general proteolysis to a compartmentalized space could increase the local concentration of the Clp protease components, thereby possibly enhancing their specific activity.

How the HSP100/ClpP protease clusters are formed is not clear. Previously, it was shown that the formation of the full ClpCP protease complex is preceded by the oligomerization of the ClpC ATPase into a hexameric ring (Kirstein *et al*., 2006). This order of assembly is likely to occur with the other Clp proteases as well (Kress *et al*., 2007), and indeed, we have shown here that a *clpX* mutant strongly reduced the appearance of ClpP–GFP foci under normal growth conditions. Interestingly, the ATPases themselves are able to form clusters in the absence of the peptidase ClpP. Therefore, the localization of the Hsp100/Clp proteins seems to be a precondition for the localization of ClpP. But what controls the localization of Hsp100/Clp proteins is not known. Whether this process requires the presence of adaptor proteins with substrates, as demonstrated *in vitro* only for one of the proteases ClpCP (Kirstein *et al*., 2006), will be difficult to test *in vivo* due to the sheer number of potential substrates and the possible existence of unknown proteins acting as adaptor proteins (Kirstein *et al*., 2007). An interesting observation was the clustering of McsB–GFP in a *clpC* knockout. Possibly, this adaptor protein can interact with one of the other Clp ATPases in the absence of ClpC, although we cannot rule out the possibility that clustering and localization are triggered in another way.

An important unresolved question with respect to the foci is not only why and how these proteases cluster but also why they cluster preferentially in the vicinity of the cell pole. A simple solution would be the presence of specific scaffolding proteins that would group the protein complexes together, but such proteins have not been identified yet.

A recent study demonstrated that in *Escherichia coli* cells protein aggregates first appear at mid-cell and the poles. During growth and subsequent cell divisions the aggregates partition to the old pole, which could explain their involvement in aging (Stewart *et al*., 2005; Lindner *et al*., 2008). Recent experiments demonstrated that aging also affects *B. subtilis* cells in a comparable manner as *E. coli* cells (Veening *et al*., 2008), suggesting that the old poles of *B. subtilis* cells are also markers for cell age and could therefore also be the subcellular place where protein aggregates might eventually accumulate.

More foci appear under heat stress, when more protein aggregates are created. And proteases colocalize with inclusion bodies, which consist of aggregated proteins. Therefore, localized aggregated proteins (Lindner *et al*., 2008) could play a role in the process of foci formation and localization. Another explanation for cluster formation, which has been put forward, is the entropy-driven depletion attraction force that is present in the cytoplasm crowded with macromolecules (Ellis, 2001; Marenduzzo *et al*., 2006). This more passive mechanism could apply for higher order protease cluster but also for the bigger structures of aggregates, recognized by the proteases.

The majority of Hsp100/ClpP protease clusters are found at the poles of cells. By blocking septum formation and chromosome replication we have shown that this localization appears to depend on the position of the nucleoid and the availability of cytosolic space. Clp proteases are very large complexes measuring about 90 by 270 Angstrom (Horwich *et al*., 1999), close to half the size of ribosomes. Probably, the dense compacted DNA–RNA complex in the nucleoid poses physical constraints and disfavours protease clustering. At least in case of inclusion bodies it is not difficult to imagine that this would prevent the presence of large protein aggregates in the area occupied by the nucleoid. The polymerization of FtsZ into the Z-ring, the initial step in septum formation, is also inhibited by the nucleoid. This so called nucleoid occlusion process is regulated (Wu and Errington, 2004; Bernhardt and de Boer, 2005; Haeusser and Levin, 2008). In our case the effect of the nucleoid seems to be more passive and to distinguish this from the former phenomenon we prefer to use the term nucleoid exclusion as part of the possible mechanism of protease localization.

Experiments with fluorescently labelled ribosomes demonstrated that they are preferentially located in the cellular space between the nucleoid and the poles of *B. subtilis* cells (Lewis *et al*., 2000; Mascarenhas *et al*., 2001). As it is widely accepted that protein synthesis requires protein quality control by chaperone systems in cooperation with proteases (Hartl and Hayer-Hartl, 2002; Bukau *et al*., 2006), it is reasonable to assume that a spatial proximity of protein synthesis and protein quality control would be advantageous for protein homeostasis and cellular function. Possibly, the clustering of Clp complexes into protease assemblies helps to separate the process of protein synthesis from that of protein degradation.

## Experimental procedures

### General methods

Cells were grown in LB medium at 30°C. chloramphenicol (10 μg ml^−1^), kanamycin (5 μg ml^−1^), tetracycline (12.5 μg ml^−1^), spectinomycin (100 μg ml^−1^), phleomycin (0.4 μg ml^−1^), erythromycin (5 μg ml^−1^) and IPTG (0.5 mM) were added as required. Heat shock was applied for 10 min at 50°C and oxidative stress was induced by the addition of 1 mM diamide to exponentially growing cells. Inhibition of protein synthesis was carried out by the addition of chloramphenicol (200 μg ml^−1^). For the thermotolerance experiment a 10 min heat shock of 42°C was applied prior to the 50°C heat shock.

Depletion of FtsZ or DnaA was performed in strains encoding *ftsZ* or *dnaA* under control of the IPTG-inducible P*spac* promoter. DNA manipulations were carried out using standard methods (Sambrook and Russell, 2001). *B. subtilis* strains harbouring the PorA expression plasmid were grown into late exponential to early stationary phase before analysis (Jürgen *et al*., 2001). Immunoblotting was performed according to standard protocols using polyclonal antibody to ClpP, CtsR, McsB [gift of U. Gerth (University of Greifswald, Germany)] and GFP (Alexis Biochemicals, Lörrach, Germany).

### Construction of fluorescent protein fusions

To create C-terminal fusions of genes encoding ClpC, ClpP, ClpX, CtsR and McsB with *gfp*, *yfp* and *cfp*, the 400 bp of the 3′ terminus were amplified by PCR and each gene fragment was cloned into the KpnI and EcoRI (*clpC*, clpE, *clpP* and *ctsR*) or KpnI and XhoI (*clpX* and *mcsB*) sites of pSG1151 (*gfp*), pSG1186 (*cfp*) and pSG1187 (yfp) respectively (Lewis and Marston, 1999; Feucht and Lewis, 2001). The construction a pSG1154/cfp (L.J. Wu, unpublished) encoding for a C-terminal *clpX–cfp* fusion was carried out using In-Fusion Dry-Down PCR Cloning Kit (Clontech) following the manufacturer's instructions. These constructs were then transformed and integrated into the respective chromosomal loci of *B. subtilis* 168 via a single or double cross-over event.

For the construction of the dually labelled strains, JK10 (*clpP–yfp clpC–cfp*) and JK28 (*clpE–yfp clpC–cfp*), the chloramphenicol resistance of JK02 (*clpC–cfp*) was changed to spectinomycin through transformation and homologous recombination using the plasmid ECE74 (pCm::Spc; Steinmetz and Richter, 1994). The strains JK04 (*clpP–yfp*), JK19 (*clpE–yfp*) and HS12 (*clpX–cfp*) were then transformed with chromosomal DNA from the resulting strain JK07 (*clpC–cfp* Spc) and selected for Cm (10 μg ml^−1^) and Spc (100 μg ml^−1^) resistance. The strains HS10 and HS11 were constructed by transforming chromosomal DNA of strain JK03 into the *B. subtilis* strains 1801 and PL20. The strains JK20–28 were constructed by transforming chromosomal DNA of the respective GFP fusion strains into the *B. subtilis* strain IH6627 carrying a plasmid harbouring the *porA* gene from *N. meningitides*. As a control the fusion were also introduced into strain IH7282 that contains the plasmid without the *porA* gene (Nurminen *et al*., 1992; Jürgen *et al*., 2001). All strains used in this study are listed in Table 1 and the oligonucleotides in Table 3.

**Table 3:** Primer for the construction of the fusion proteins.

Name	Sequence
ClpC–GFP-for	GGGGTACCAACTCAGAGGTTACTGTGGATG
ClpC–GFP-rev	GGGAATTCATTCGTTTTAGCAGTCGTTTTTACG
ClpX–YFP-for	GGGGTACCGTTGCTGTGTATAACCAC
ClpX–YFP-rev	CCGCTCCAGTGCAGATGTTTTATCTTGGCTTACC
ClpE–YFP-for	GGGGTACCGCTCATACCGCTGTCACTG
ClpE–YFP-rev	GGGAATTCTTTTGCTCGCACTTTGATTTTATC
ClpP–GFP-for	GGGGTACCATCAACAGCCCGGGCGGCTC
ClpP–GFP-rev	GGGAATTCCTTTTTGTCTTCTGTGTGAGTC
McsB–YFP-for	GGGGTACCATACGTTTAGCAAGAAACTTTGAGC
McsB–YFP-rev	CCGCTCGAGTATCGATTCATCCTCCTGTC
CtsR–YFP-for	GGGGTACCGGCAAGGAAATTTTAGAG
CtsR–YFP-rev	GGGAATTCTTTTAATTTTAAAGAAGTC
ClpX–CFP-for	GAGATTCCTAGGGGGGCAATATAGTTAATGCAGG
ClpX–CFP-rev	ACCGTCGACCTCGAGTGCAGATGTTTTATCTTGGCTTACC
pSG1154/CFP-for	CATCCTAGGAATCTCCTTTCTAG
pSG1154/CFP-rev	CTCGAGGTCGACGGTATCGATAAG

### Fluorescence microscopy

Samples were treated as described, withdrawn and cells were mounted on agarose-covered microscope slides (SM-011, Hendley Essex) and examined with an Axiovert 135TV microscope (Zeiss). Images were processed using the metamorph V5.0 software (Universal Imaging, Media, PA, USA), softWoRx Suite (Applied Precision) and ImageJ v.1.38 (NIH). The subcellular localization of the fluorescent proteins was further examined by the additional staining of the nucleoid with 1 μg ml^−1^ DAPI (Sigma) and membrane by addition of 1 μl of Nile Red (100 μg ml^−1^; Sigma) to 100 μl culture samples.

### Immunofluorescence

Heat-shocked cells were fixed in PBS containing 16% paraformaldehyde and 25% glutaraldehyde for 15 min at room temperature followed by 30 min on ice. The cells were then washed three times in PBS and re-suspended in 50 mM glucose, 20 mM Tris pH 7.5, 10 mM EDTA and spotted onto microscopic slides. For permeabilization of the cells, lysozyme was added to a final concentration of 2 mg ml^−1^ for 1 min. Cells were then washed and blocked with 2% BSA in PBS buffer. The primary ClpP antibody was used in a dilution of 1:20 000 and incubated over night at 4°C. Cells were extensively washed with PBS and incubated with the secondary antibody overnight at 4°C. Two microlitres of antifade was added to each well and slides were then covered and sealed for microscopic inspection.

### Time-Lapse microscopy

Time-Lapse microscopy was essentially carried out as described (Veening *et al*., 2008). For Movies S1–S3, microscope slides were incubated at 30°C in a temperature-controlled Deltavision RT automated microscope (Applied Precision). Images were obtained with a CoolSNAP HQ (Princeton Instruments) at a magnification of 63×. Fluorescent images were recorded every 8 min (Movies S1 and S2) or 1 min (Movie S3). For Movie S3, LB medium was used instead of the medium described (Veening *et al*., 2008). To prevent phototoxicity, the excitation light (480–500 nm, 0.75 s in Movies S1 and S2, 1 s in Movie S3) was limited to 32% (Movies S1 and S2) or 1% (Movie S3) of the output of a 100 W Hg-vapour lamp by neutral density filters. Used emission wavelengths for GFP were 509–547 nm (filters from Chroma). Movies were generated using softWoRx Suite (Applied Precision) and ImageJ v.1.38 (NIH).

### Supporting information

Time-lapse movies of growing microcolonies of *B. subtilis clpP–gfp*. Movie S1 shows the outgrowth of a whole colony with pictures taken every 8 min. Movie S2 shows a detail of Movie S1. Movie S3 shows a time-lapse movie of a faster growing microcolony with pictures taken every minute. See *Experimental procedures* for a detailed description.
