# Supplementary material for: Localization of general and regulatory proteolysis in Bacillus subtilis cells
Source: Mol Microbiol. 2008 Sep 29;70(3):682–94. doi: 10.1111/j.1365-2958.2008.06438.x (PMC2628427; doi:10.1111/j.1365-2958.2008.06438.x)
Supplement: Supplementary file 1 [file mmi0070-0682-SD1.pdf]

Supporting Information:

Fig. S1: Heat shock induced **l**ocalization of ClpP in the absence of *de novo* protein synthesis.

The ClpP localization is visualized in *wild type B. subtilis* strain at 30°C (left panel), after heat shock in 50°C (middle panel) and after heat shock and simultaneous inhibition of *de novo* protein synthesis with chloramphenicol (right panel).

Fig. S2: Colocalization Clp proteases after heat shock.

Dual labeling of ClpE-ClpX, ClpE-ClpC, ClpC-ClpP and ClpX-ClpP at 50°C. The images were obtained using YFP filters (left column) and CFP filters (middle column). An overlay of the YFP and CFP channels is shown on the right. YFP fluorescence has been pseudocoloured in red and CFP in green.

Fig. S3: Cellular localization of ClpP in the absence of replication and *de novo* protein synthesis.

The ClpP localization is visualized in a DnaA depleted *B. subtilis* strain at 30°C (upper panel), after heat shock in 50°C (middle panel), and after heat shock and simultaneous inhibition of *de novo* protein synthesis with chloramphenicol (lower panel). Phase contrast (i), membrane stain (ii), nucleoid stain (iii), GFP fluorescence (iv), and the merged images (v) are subsequently depicted. (see experimental procedures for details).

Fig. S4: Heat shock induction of McsB and CtsR

Western blot of cytosolic extracts from normal growing and heats hocked cells. The tested mutant is indicated on the left and the detected protein is depicted on the right. In these blots antisera against CtsR and McsB were used, but Anti-GFP sera gave similar results.

Supporting Fig. S1

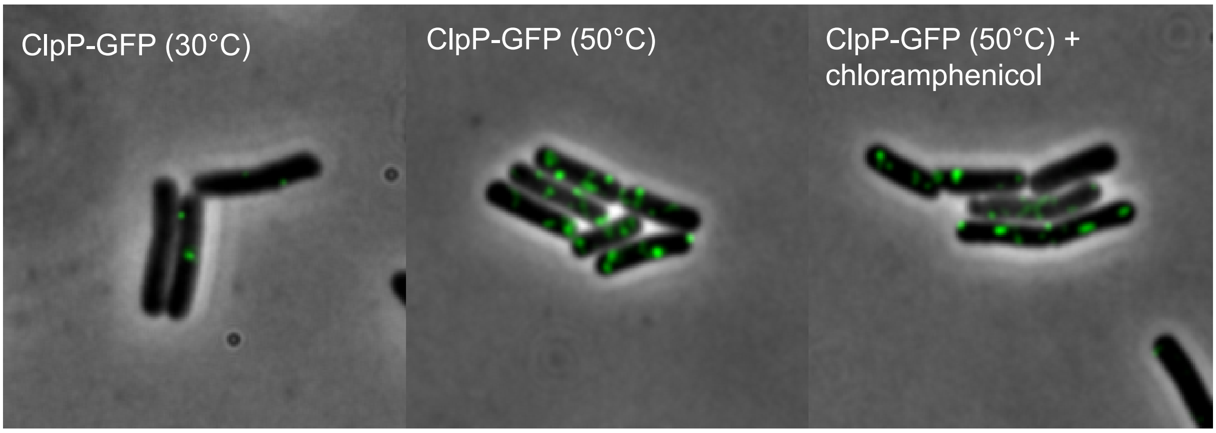

Supporting Fig. S2

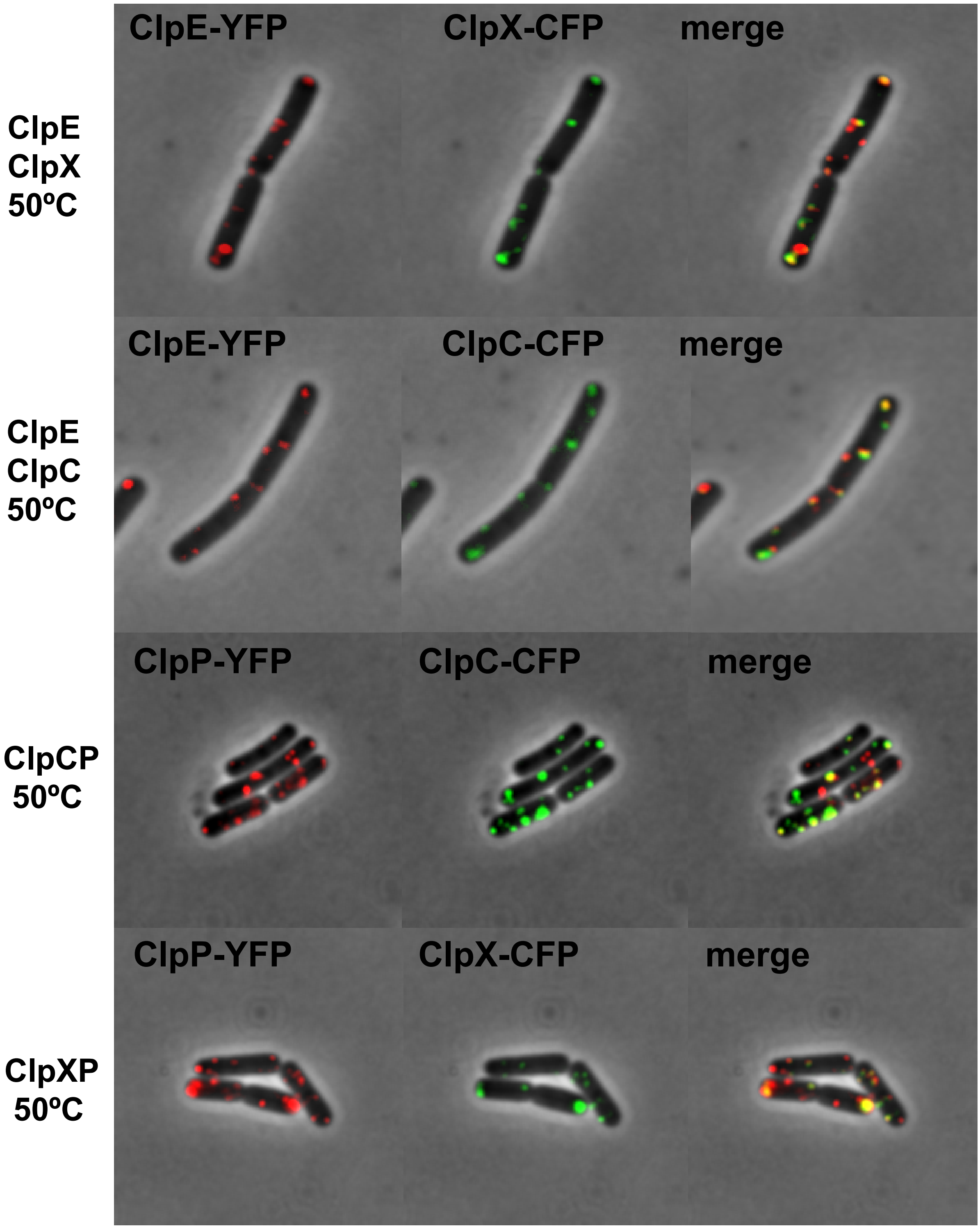

Supporting Fig. S3

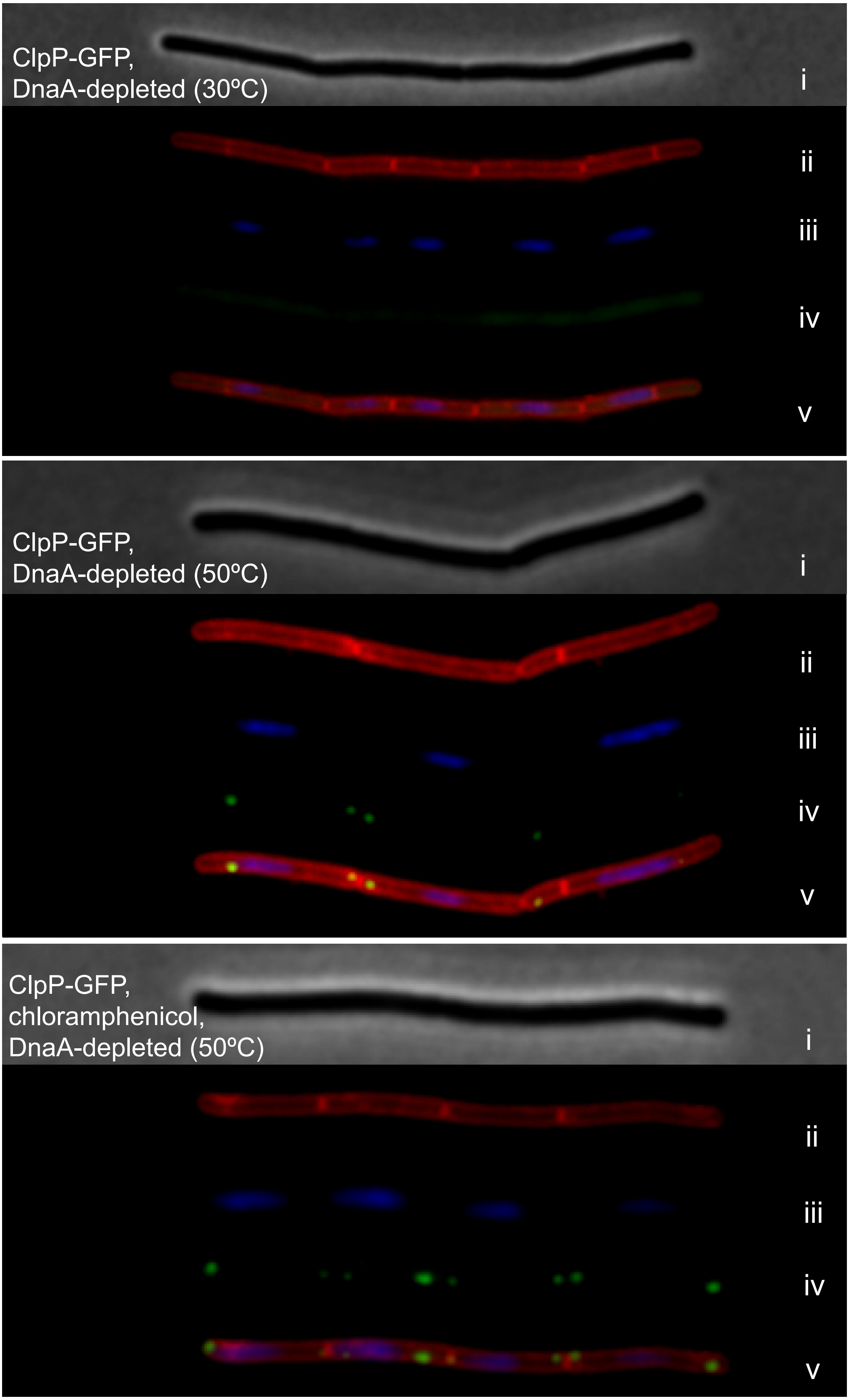

supporting Fig. S4

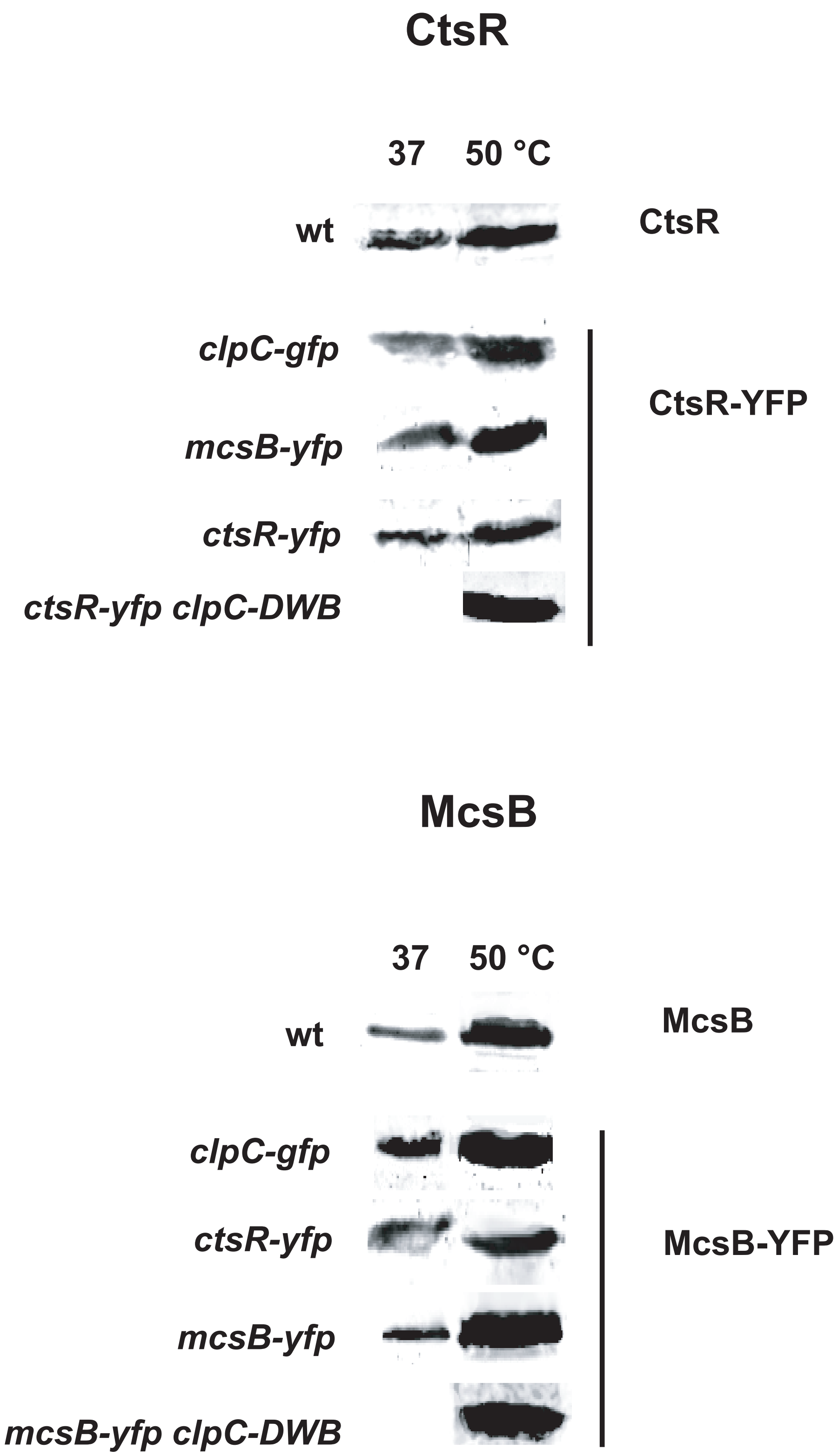

Movie S1: Growth of *B. subtilis* microcolony encoding ClpP-GFP. This movie displays 12h of the growth of a *B. subtilis* microcolony with ClpP-GFP used for visualization of the dynamic localization of ClpP. Individual frames of the movie were obtained in time interval of 8 minutes. Data derived from this microcolony were used in Fig. 1B, Movie S2 and statistical analysis.

Movie S2: Detailed movie visualizing dynamic localization of ClpP-GFP in *B. subtilis.* This movie was derived from Movie S1 (time interval of 8 minutes). Individual frames are also depicted in Fig. 1B.

Movie S3: Growth of *B. subtilis* encoding ClpP-GFP in LB medium. This movie displays 1.5h of the growth of *B. subtilis* with ClpP-GFP used for visualization of the dynamic localization of ClpP. Individual frames of the movie were obtained in time intervals of 1 minute.
